# Associations between COVID-19 testing status, non-communicable diseases and HIV status among residents of sub-Saharan Africa during the first wave of the pandemic

**DOI:** 10.1186/s12879-022-07498-w

**Published:** 2022-06-13

**Authors:** Morenike O. Folayan, Roberto Ariel Abeldaño Zuñiga, Jorma I. Virtanen, Maha El Tantawi, Giuliana Florencia Abeldaño, Anthonia Omotola Ishabiyi, Mohammed Jafer, Nuraldeen Maher Al-Khanati, Mir Faeq Ali Quadri, Muhammad Abrar Yousaf, Passent Ellakany, Ntombifuthi Nzimande, Eshrat Ara, Zumama Khalid, Folake Barakat Lawal, Joanne Lusher, Bamidele O. Popoola, Ifeoma Idigbe, Abeedha Tu-Allah Khan, Martin Amogre Ayanore, Balgis Gaffar, Bamidele Emmanuel Osamika, Nourhan M. Aly, Nicaise Ndembi, Annie Lu Nguyen

**Affiliations:** 1 Mental Health and Wellness Study Group, Ile-Ife, Nigeria; 2 Department of Child Dental Health, Obafemi Awolowo University, Ile-Ife, Nigeria; 3 Postgraduate Department, University of Sierra Sur, Oaxaca, Mexico; 4 Faculty of Medicine, University of Turku, Turku, Finland; 5 Department of Pediatric Dentistry and Dental Public Health, Faculty of Dentistry, Alexandria University, Alexandria, 21527 Egypt; 6 School of Medicine, University of Sierra Sur, Oaxaca, Mexico; 7 Centre for Rural Health, School of Nursing and Public Health, University of KwaZulu-Natal, Durban, South Africa; 8 Department of Preventive Dental Sciences, College of Dentistry, Jazan University, Jazan, Saudi Arabia; 9 Department of Health Promotion, Faculty of Health, Medicine and Life Sciences, Maastricht University, Maastricht, The Netherlands; 10 Department of Oral and Maxillofacial Surgery, Faculty of Dentistry, Syrian Private University, Damascus, Syria; 11 Dental Public Health, College of Dentistry, Jazan University, Jazan, Saudi Arabia; 12 Department of Biology, Virtual University of Pakistan, Lahore, Pakistan; 13 Department of Substitutive Dental Sciences, College of Dentistry, Imam Abdulraman Bin Faisal University, Dammam, Saudi Arabia; 14 Department of Economic and Social Geography, Faculty of Science and Informatics, University of Szeged, 6722 Szeged, Hungary; 15 Department of Psychology, Government College for Women, Moulana Azad Road, Srinagar, Kashmir (J&K) India; 16 Department of Health Sciences, University of Genoa, 16132 Genoa, Italy; 17 Department of Periodontology and Community Dentistry, University of Ibadan and University College Hospital, Ibadan, Nigeria; 18 Regent’s University London, London, UK; 19 Department of Child Oral Health, University of Ibadan, Ibadan, Nigeria; 20 Clinical Sciences Department, Nigerian Institute of Medical Research, Lagos, Nigeria; 21 School of Biological Sciences, University of the Punjab, New Campus, Lahore, 54590 Punjab Pakistan; 22 Department of Health Policy Planning and Management, University of Health and Allied Sciences, Ho, Ghana; 23 Preventive Dental Sciences Department, College of Dentistry, Imam Abdulrahman Bin Faisal University, Dammam, Saudi Arabia; 24 Department of Psychology, Miami University, Oxford, OH USA; 25 Africa Centres for Disease Control and Prevention, African Union Commission, Roosevelt Street, Addis Ababa, Ethiopia; 26 Department of Family Medicine, Keck School of Medicine, University of Southern California, Los Angeles, CA USA

**Keywords:** Respiration disorder, Depression, HIV, COVID-19, COVID-19 testing

## Abstract

**Background:**

This study determined if non-communicable disease status, HIV status, COVID-19 status and co-habiting were associated with COVID-19 test status in sub-Saharan Africa.

**Methods:**

Data of 5945 respondents age 18-years-old and above from 31 countries in sub-Saharan Africa collected through an online survey conducted between June and December 2020, were extracted. The dependent variable was COVID-19 status (testing positive for COVID-19 and having symptoms of COVID-19 but not getting tested). The independent variables were non-communicable disease status (hypertension, diabetes, cancer, heart conditions, respiratory conditions, depression), HIV positive status, COVID-19 status (knowing a close friend who tested positive for COVID-19 and someone who died from COVID-19) and co-habiting (yes/no). Two binary logistic regression models developed to determine associations between the dependent and independent variables were adjusted for age, sex, employment, sub region and educational status.

**Results:**

Having a close friend who tested positive for COVID-19 (AOR:6.747), knowing someone who died from COVID-19 infection (AOR:1.732), and living with other people (AOR:1.512) were significantly associated with higher odds of testing positive for COVID-19 infection, while living with HIV was associated with significantly lower odds of testing positive for COVID-19 infection (AOR:0.284). Also, respondents with respiratory conditions (AOR:2.487), self-reported depression (AOR:1.901), those who had a close friend who tested positive for COVID-19 infection (AOR:2.562) and who knew someone who died from COVID-19 infection (AOR:1.811) had significantly higher odds of having symptoms of COVID-19 infection but not getting tested.

**Conclusion:**

Non-communicable diseases seem not to increase the risk for COVID-19 positive test while cohabiting seems to reduce this risk. The likelihood that those who know someone who tested positive to or who died from COVID-19 not getting tested when symptomatic suggests there is poor contact tracing in the region. People with respiratory conditions and depression need support to get tested for COVID-19.

**Supplementary Information:**

The online version contains supplementary material available at 10.1186/s12879-022-07498-w.

## Introduction

Sub-Saharan Africa is a low to middle income region with a high prevalence of communicable diseases [1], HIV infection [2, 3] and an increasing prevalence of non-communicable diseases [4, 5]. It is expected that about 27% of mortality related to non-communicable diseases in Africa will occur in the sub-Saharan Africa region [6].

The number of persons in sub-Saharan Africa who tested positive for COVID-19 has been low, and attributable to the low rate of COVID-19 testing [7]. Many countries in this region have few laboratories and trained personnel to conduct and maintain calibrated Real-Time Reverse Transcription Polymerase Chain Reaction equipment, which is the gold standard for detecting SARS-CoV-2 [8]. In addition, countries in the region have insufficient resources to provide the continuous supply of essential reagents needed for COVID-19 screening [9]. Therefore, multiple asymptomatic cases go undetected and symptomatic cases may be mistaken for mild upper respiratory diseases [10]. There is also widespread of misinformation and misconceptions about COVID-19 which instigates stigma and keeps people away from being tested [11], and there is poor access to testing services and care especially in the rural area [12].

The region also has a very low coverage of COVID-19 vaccination [13], and together with the low rate of testing and detection of symptomatic and asymptomatic cases, the risk for multiple waves of the COVID-19 pandemic increases. It is therefore essential to identify populations at high risk of COVID-19 infection and prioritise efforts to increase their access to COVID-19 infection prevention information and COVID-19 vaccination.

Prior studies have indicated that persons living with HIV are at higher risk of contracting and dying from COVID-19 infection [14, 15] as both diseases have common biological, clinical, and epidemiological factors that affect the acquisition and clinical impact of these infections [16]. Also, people living with HIV are less likely to be vaccinated against COVID-19 [17] though vaccination uptake was better among those with chronic diseases [18]. The severity of COVID-19 and death from the disease are higher in those who are not vaccinated [19] and those who have COVID-19 related symptoms and delayed access to care [20]. COVID-19 vaccine hesitancy may also be linked to poor uptake of COVID-19 tests.

Moreover, people living with certain non-communicable diseases like obesity [21], respiratory disorders [22], diabetes [23], cancer [24], depression [25], cardiovascular diseases, and heart conditions [26] are at higher risk of death from COVID-19 infection. The risk profile for COVID-19 infection also differs by age, sex, educational status, employment status and residential status. Younger respondents have been less impacted by testing positive and being symptomatic for COVID-19 [27], while males are at greater risk of contracting, and dying from severe COVID-19 infection [28]. The educational status also affects the perception of risk and adherence to COVID-19 protective behaviours [29] while employment status has indirect associations with COVID-19 risk as this affects the mental health status and financial security of individuals [30].

There is little information on the factors associated with the uptake of COVID-19 tests by people in sub-Saharan Africa living with non-communicable diseases and HIV infection. A prior study conducted in Nigeria indicated that significantly fewer people living with HIV tested positive for COVID-19 infection than those not living with HIV [31]. Also, patients with hypertension, cancer, diabetes, or HIV infection had a higher risk of dying from COVID-19 infection in Nigeria [32]. We were unable to obtain data on the risk profile for COVID-19 positivity tests in sub-Saharan Africa. Also, no studies identifying factors associated with not taking a COVID-19 test despite having symptoms of COVID-19 infection were found.

The aim of this study was to determine the factors associated with COVID-19 test status among residents in sub-Saharan Africa during the first wave of the COVID-19 pandemic. Specifically, the factors investigated were having a non-communicable disease, living with HIV, knowing someone with COVID-19 infection or who died from COVID-19 infection, and living arrangement. We hypothesised that people in sub-Saharan Africa living with non-communicable diseases or HIV will have higher odds of testing positive for COVID-19 than respondents who are not living with non-communicable diseases or HIV. Furthermore, we hypothesised that respondents who had a close friend who had tested positive for COVID-19 infection and who knew someone who died from COVID-19 may be at increased risk of exposure to COVID-19 infection and thus are more likely to have symptoms of COVID-19 infection and test positive for COVID-19. However, respondents who cohabitate are likely to be more careful and avoid putting others at risk for COVID-19 infection and are, therefore, less likely to test positive for COVID-19 infection.

## Methods

### Ethics approval and consent to participate

The study protocol was approved by the Human Research Ethics Committee at the Institute of Public Health, Obafemi Awolowo University, Ile-Ife, Nigeria (IPHOAU/12/1557). Informed consent was obtained from the study participants for the online survey by asking them to tick a checkbox that indicated consenting to study participation. Study participants could only proceed to the survey having ticked the checkbox. The study was performed in accordance with the National Health Research Ethics Code. All methods were carried out in accordance with National Health Research Ethics Code.

### Study design, study participants and study participants’ recruitment

The data for this cross-sectional study was extracted from a multi-country survey on the mental health and wellness of a global convenience sample of adults aged 18 years and older collected during the first wave of the pandemic (June 29 to December 31, 2020) [33]. There were no exclusion criteria. Data were collected from participants recruited through respondent-driven sampling using an online survey platform (SurveyMonkey®). Initially, 45 data collectors shared the survey link through their networks within and outside their country of residence using the social media (Facebook, Twitter, and Instagram), network email lists, and WhatsApp groups.

The data collection tool was developed for a study targeting a specific population in the United States [34] and was consequently adapted, translated from English to French, Portuguese, Spanish, and Arabic; and these translated versions were back-translated to English to ensure consistency of meaning (Additional file 2). The instrument underwent four iterative processes for content validation. The overall content validity index of the survey tool was 0.83. The responses collected for content validation were excluded from the final data analysis. Study participants completed an anonymous, closed-ended questionnaire preceded by a brief introduction of the study team and the objectives of the study. Each participant could only complete a single questionnaire through IP address restrictions, though they could edit their answers freely until they chose to submit. The average time of completing the survey was 11 min.

### Dependent variables

Respondents were asked if they had tested positive for COVID-19 or had COVID-19 symptoms but did not get tested. Response choices for these items were either ‘yes’ or ‘no’. This question was based on items from the mental health and wellness study [31].

### Independent variables

*History of non-communicable diseases*: Respondents were asked to identify if they had any of the 23 listed health conditions presented on a checklist in addition to other health conditions not listed. These included medical conditions which put individuals at higher risk for severe COVID-19 disease (respiratory conditions, diabetes, cancer, heart condition) and those that might put people at moderate risk of COVID-19 disease (respiratory problems, hypertension, depression) [35].

*HIV status*: As part of the 23 listed medical health conditions, participants were also asked about their HIV status. Respondents self-reported if they were living with HIV by ticking a checkbox to indicate yes. All respondents who did not tick the checkbox were categorised as not living with HIV.

*COVID-19 status*: Respondents were asked if they had a close friend who had tested positive for COVID-19 or knew someone who died from COVID-19. Response choices for these items were ‘yes’ or ‘no’ [31].

*Depression*: Respondents were asked to indicate if they had experienced depression during the pandemic by checking a response box. A check indicated that the respondent self-reported depression. The question was adapted from the Pandemic Stress Index [36].

*Co-habitation*: Respondents were asked if they were living with other people (yes, no) at the time of the survey.

### Confounders

Respondents were asked about their country of residence during the pandemic, age (in years), sex at birth, the highest level of education attained (none, primary, secondary, and tertiary), employment status (retired, student, employed, and unemployed) and the sub regions (Western and Central Africa, and Eastern and Southern Africa [37]). Only the data of respondents who resided in one of the 54 countries in sub-Saharan Africa were included in this study. Data extracted for the study were for participants representing 31 out of the 54 countries in the region. See Additional file 1 for details of countries included in the analysis.

### Data analysis

Descriptive analysis of all study variables was conducted. T-test and chi-square test were used to determine the associations between dependent, independent and confounding variables. Two binary logistic regression models were constructed to identify the independent variables significantly associated with the study dependent variables. The logistic regression models developed were adjusted for the sociodemographic status of the study participants (age at last birthday, sex at birth, employment status and educational status). The adjusted odds ratios (AOR) and 95% confidence intervals (CIs) were calculated. Statistical significance was set at less than 5%.

## Results

There were 21106 global participants who accessed the survey questionnaire of which 20083 (95.2%) consented to participate. Of the 20083 study participants, 5983 (29.8%) were from sub-Saharan Africa. Of the 5983 participants from sub-Saharan Africa, 5945 (99.4%) provided complete responses. Table 1 shows that of the 5945 participants included in the study, 167 (2.8%) reported testing positive for COVID-19 during the study period, and 649 (10.9%) had COVID-19 symptoms but did not take a COVID-19 test. Also, 139 (2.3%) had diabetes, 476 (8.0%) had hypertension, 15 (0.3%) had cancer, 46 (0.8%) had heart conditions, 57 (1.0%) had respiratory conditions, 414 (7.0%) had depression and 983 (16.5%) were living with HIV. In addition, 1192 (20.1%) had a friend who tested positive for COVID-19 infection, and 1934 (32.5%) knew someone who died from COVID-19.

**Table 1:** Factors associated with COVID-19 status by adults in sub-Saharan Africa

Variables	Total N = 5945	Tested positive for COVID-19 infection					Had symptoms of COVID-19 but did not get tested				
		No		Yes		p value	No		Yes		p value
		N = 5778		N = 167			N = 5296		N = 649		
		n	(%)	n	(%)		N	(%)	n	(%)	
Age mean (SD) in years		Mean 36.4	SD 11.4	Mean 36.9	SD 11.7	0.595	Mean 36.9	SD 11.5	Mean 32.9	SD 9.8	** < 0.001**
Sex											
Male	2745	2665	97.1	80	2.9	0.649	2409	87.8	336	12.2	**0.002**
Female	3200	3113	97.3	87	2.7		2887	90.2	313	9.8	
Level of education											
No formal education	55	53	96.4	2	3.6	0.461	49	89.1	6	10.9	0.698
Primary	89	87	97.8	2	2.2		81	91.0	8	9.0	
Secondary	887	869	98.0	18	2.0		781	88.0	106	12.0	
Tertiary	4914	2693	97.0	79	3.0		4385	89.2	529	10.8	
Employment status											
Retired	139	134	96.4	5	3.6	0.610	132	95.0	7	5.0	**0.014**
Student	784	771	98.3	13	1.7		680	86.7	104	13.3	
Employed	4130	4000	96.9	130	3.1		3696	89.5	434	10.5	
Unemployed	892	873	97.9	19	2.1		788	88.3	104	11.7	
Sub regions											
Western and Central Africa	5086	4952	97.4	134	2.6	**0.038**	4542	89.3	544	10.7	0.275
Eastern and Southern Africa	859	826	96.2	33	3.8		774	87.8	106	12.2	
Non-communicable disease											
Diabetes											
No	5806	5643	97.2	163	2.8	0.960	5172	89.1	634	10.9	0.962
Yes	139	135	97.1	4	2.9		124	89.2	15	10.8	
Hypertension											
No	5469	5325	97.4	144	2.6	**0.005**	4871	89.1	598	10.9	0.883
Yes	476	453	95.2	23	4.8		425	89.3	51	10.7	
Cancer											
No	5930	5765	97.2	166	2.8	0.365	5285	89.1	645	10.9	0.050
Yes	15	14	93.3	1	6.7		11	73.3	4	26.7	
Heart conditions											
No	5899	5736	97.2	163	2.8	**0.015**	5260	89.2	639	10.8	**0.018**
Yes	46	42	91.3	4	8.7		36	78.3	10	21.7	
Respiratory conditions											
No	5888	5723	97.2	165	2.8	0.748	5256	89.3	632	10.7	** < 0.001**
Yes	57	55	96.5	2	3.5		40	70.2	17	29.8	
Depression											
No	5531	5379	97.3	152	2.7	0.299	4968	89.8	563	10.2	** < 0.001**
Yes	414	399	96.4	15	3.6		328	79.2	86	20.8	
HIV status											
Not living with HIV	4962	4804	96.8	158	3.2	** < 0.001**	4414	89.0	548	11.0	0.480
Living with HIV	983	974	99.1	9	0.9		882	89.7	101	10.3	
COVID-19 status											
Friend tested positive for COVID-19 infection											
No	4753	4695	98.8	58	1.2	** < 0.001**	4351	91.5	402	8.5	** < 0.001**
Yes	1192	1083	90.9	109	9.1		945	79.3	247	20.7	
Know someone who died of COVID-19 infection											
No	4011	3942	98.3	69	1.7	** < 0.001**	3667	91.4	344	8.6	** < 0.001**
Yes	1934	1836	94.9	98	5.1		1629	84.2	305	15.8	
Co-habitation											
No	1296	1248	97.4	48	2.6	**0.028**	1143	89.3	153	10.7	0.246
Yes	4649	4530	96.3	119	3.7		4153	88.2	496	11.8	

Table 1 also shows that significantly more respondents living in Eastern and Southern Africa sub region (p = 0.038), who had hypertension (p = 0.005), who had heart conditions (p = 0.015), who had a friend who tested positive for COVID-19 (p < 0.001), who knew someone who died from COVID-19 infection (p < 0.001) and who were cohabiting (p = 0.028) tested positive for COVID-19 infection. Also, significantly fewer respondents who were HIV positive (p < 0.001) tested positive for COVID-19.

In addition, significantly younger people (p < 0.001), more males than females (p = 0.002); more students compared to other professions (p = 0.014); people with heart conditions (p = 0.018), respiratory conditions (p < 0.001), and self-reported depression (p < 0.001); and respondents who had a friend who tested positive for COVID-19 (p < 0.001) and who knew someone who died from COVID-19 infection (p < 0.001) had COVID-19 symptoms but did not get tested.

Table 2 highlights that there was no significant association between non-communicable diseases and testing positive for COVID-19 infection. Respondents who had a close friend who tested positive for COVID-19 infection (AOR: 6.747; 95% CI: 4.730–9.622; p < 0.001); those who knew someone who died from COVID-19 infection (AOR: 1.732; 95% CI: 1.231–2.437; p = 0.002); and respondents living with other people (AOR: 1.512; 95% CI: 1.058–2.162; p = 0.023) had significantly higher odds of testing positive for COVID-19 infection. People living with HIV had significantly lower odds of testing positive for COVID-19 infection (AOR: 0.284; 95% CI: 0.129–0.622; p = 0.002).

**Table 2:** Binary logistic regression to determine factors the association between HIV testing status, COVID-19 status non-communicable disease and HIV status for residents of sub-Saharan Africa (N = 5945)

Variables	Tested positive for COVID-19 infection				Had symptoms of COVID-19 but did not get tested			
	AOR	95% C.I. for AOR		P value	AOR	95% C.I. for AOR		P value
		Lower	Upper			Lower	Upper	
Age	0.991	0.973	1.009	0.334	0.955	0.945	0.965	< 0.001
Males (Ref: females)	0.986	0.714	1.362	0.931	1.377	1.160	1.635	< 0.001
Education (Ref: No formal education)	1. 000	–	–	–	1. 000	–	–	–
Primary	0.890	0.107	7.377	0.914	0.688	0.212	2.238	0.535
Secondary	0.458	0.086	2.440	0.360	0.936	0.367	2.384	0.890
Tertiary (University)	0.290	0.057	1.475	0.136	0.922	0.367	2.315	0.862
Employment status (Ref: unemployed)	1. 000	–	–	–	1. 000	–	–	–
Retired	2.275	0.691	7.488	0.176	1.345	0.563	3.210	0.504
Students	0.559	0.260	1.202	0.137	0.891	0.645	1.231	0.485
Employed	0.991	0.585	1.677	0.972	0.993	0.774	1.273	0.956
*Sub region*								
Eastern and Southern Africa (ref: Western and Central Africa)	0.817	0.537	1.242	0.344	0.760	0.596	0.969	0.027
*Medical health profile*								
Diabetes (ref: no)	0.669	0.226	1.983	0.469	1.120	0.614	2.044	0.711
Hypertension (ref: no)	1.573	0.930	2.661	0.091	1.201	0.849	1.699	0.301
Cancer (ref: no)	0.680	0.071	6.523	0.738	1.335	0.324	5.496	0.689
Heart condition (ref: no)	1.605	0.508	5.070	0.420	1.947	0.884	4.290	0.098
Respiratory condition (ref: no)	0.830	0.193	3.573	0.802	2.487	1.348	4.591	0.004
Depression (ref: no)	1.115	0.618	2.011	0.719	1.901	1.442	2.508	< 0.001
Living with HIV (ref: no)	0.284	0.129	0.622	0.002	1.280	0.982	1.670	0.068
*COVID-19 status*								
I have a close friend who tested positive for COVID-19 (ref: no)	6.747	4.730	9.622	< 0.001	2.562	2.113	3.107	< 0.001
I know someone who died from COVID-19 (ref: no)	1.732	1.231	2.437	0.002	1.811	1.510	2.172	< 0.001
Cohabiting (Ref: no)	1.512	1.058	2.162	0.023	1.020	0.833	1.249	0.850

In addition, male respondents (AOR: 1.377; 95% CI: 1.160–1.635; p < 0.001), respondents with respiratory conditions (AOR: 2.487; 95% CI: 1.348–4.591; p = 0.004) and self-reported depression (AOR: 1.901; 95% CI: 1.442–2.508; p < 0.001), respondents who had a close friend who tested positive for COVID-19 infection (AOR: 2.562; 95% CI: 2.113–3.107; p < 0.001) and respondents who knew someone who died from COVID-19 infection (AOR: 1.811; 95% CI: 1.510–2.172; p < 0.001) had significantly higher odds of having symptoms of COVID-19 infection and not getting tested. Also, younger respondents (AOR: 0.955; p < 0.001) and participants from the Eastern and Southern sub region of Africa (AOR: 0.760; 95% CI: 0.596–0.969; p = 0.027) had significantly lower odds of having symptoms of COVID-19 infection and not getting tested. Data shown in Additional file 3 indicates that residents in Southern (AOR: 0.738; 95% CI: 0.567–0.960; p = 0.023) and not Eastern (AOR: 0.885; 95% CI: 0.514–1.522; p = 0.658) Africa had the significantly lower odds of having symptoms of COVID-19 infection and not getting tested.

## Discussion

The study found that respondents with non-communicable diseases did not have higher odds of testing positive for COVID-19 compared with those without non-communicable diseases. However, respondents with respiratory conditions and depression had significantly higher odds of not getting tested for COVID-19 infection despite having symptoms. On the other hand, respondents living with HIV had significantly lower odds of testing positive for COVID-19 but insignificantly higher odds of not getting tested for COVID-19 when they had symptoms. Respondents who had a friend who tested positive for COVID-19 and knew someone who died from COVID-19 infection had higher odds of testing positive for COVID-19 and having COVID-19 symptoms and not getting tested. Finally, respondents living with others had significantly higher odds of testing positive for COVID-19 while respondents living in Southern Africa had significantly lower odds of having COVID-19 symptoms and not getting tested. These study results partially support the study hypotheses.

One of the strengths of the study is the contribution to understanding the epidemiology of the COVID-19 pandemic in sub-Saharan Africa, a region with a high prevalence of HIV and a growing prevalence of non-communicable diseases. This study provides information about the COVID-19 related behavioural responses of persons with HIV infection and those with non-communicable diseases, which helps to better understand the biological, clinical, and epidemiological relationship between both infections [16]. The large sample size and the representation of all the sub-regions in sub-Saharan Africa make the findings reliable. However, a higher proportion of respondents from Nigeria, South Africa and Ghana, the data was collected using convenient sampling and the skewness of the study participants to those with tertiary education, limits the generalizability of the study findings. In addition, this is a cross-sectional study and thus causality cannot be deduced from the study findings.

Despite the limitations of the study, the findings highlighted some important information that can guide policy makers on the potential directions for further research and COVID-19 care measures. First, people who had friends with a history of COVID-19 infection and those who knew people who died from COVID-19 had significantly higher odds of a COVID-19 positive test and having COVID-19 symptoms but not getting tested. This implies that the surveillance system in the region may be weak resulting in poor contact tracing and case detection and increased risk of ongoing COVID-19 transmission within the community as earlier noted [38]. The surveillance system and contact tracing in the region could be strengthened to allow for prompt detection of disease and subsequent isolation/quarantine as a strategy for reducing transmission.

Second, stigma and discrimination may be a concern in the region and a reason why people with COVID-19 symptoms do not getting tested. This is reflected in the study findings that indicates that people living with HIV had higher odds of having symptoms for COVID-19 and not getting tested; and lower odds of testing positive for COVID-19 infection. Concerns about stigma and discrimination for a positive COVID-19 result are salient [39] especially for people living with HIV who have had to deal with stigma and discrimination from being HIV positive [40]. These study results indicating that people living with HIV are at low risk for COVID-19 infection in the region should therefore, be interpreted with caution. We postulate that people living with HIV who are likely to be infected may not be getting tested because of multiple concerns including having to isolate/quarantine and thereby having challenges accessing their antiretroviral therapy especially if they had lived discretely with their HIV status. Therefore, people living with HIV in the region need more support to get tested for COVID-19 to reduce the risk for severe morbidity and mortality associated with the infection. It may be possible that access to COVID-19 self-testing kits may improve the uptake of COVID-19 testing by people living with HIV where stigma and the need for confidential testing may have deterred some from taking a test. Also, support for home management of infections may reduce the risk of the stigma associated with isolation/quarantine in facilities as well as possible antiretroviral therapy access while being isolated/quarantined.

Third, people with depression were more likely to have COVID-19 symptoms without getting tested. The pandemic itself is a risk factor for depression [41], and depression is also a risk factor for COVID-19 infection and death [25]. Failure to get tested therefore, despite having the symptoms of COVID-19, may lead to high mortality in COVID-19 infected persons who are depressed. Apathy associated depression may increase the risk for being careless with self and therefore, not taking a test even when one has symptoms of COVID-19 infection. Further studies are necessary to better understand the reasons why those who feel depressed are less likely to take a COVID-19 test when they experience symptoms of COVID-19.

Fourth, respondents in the present study who reported respiratory conditions had higher odds of symptoms without getting tested for COVID-19. The reasons for this remain unclear. Symptoms of COVID-19 may be misdiagnosed as respiratory disorders [42]. Having a high index of suspicion for COVID-19 should promote screening for COVID-19 among people with respiratory conditions. Further studies are needed to explore reasons for low COVID-19 testing among those with respiratory disorders in the region despite experiencing symptoms of COVID-19 infection.

Finally, demographic factors like living arrangements, age, sex, and region of residence were significantly associated with COVID-19 status. We observed that people who live with others had higher odds of testing positive for COVID-19. This may be because people who live in the same close space are less likely to use COVID-19 protective measures like face masks and social distancing thereby increasing the risk for cross infections especially when the ventilation is poor [43]. This study results provides one more evidence to justify the need to continue to research on how to improve ventilation as this may yield benefits during the next pandemic [43].

Also, we observed that younger respondents are less likely to have symptoms and take a COVID-19 test. Older people have a higher risk of co-morbidities and contracting COVID-19 infection and dying from the infection [44]. Therefore, older people are more likely to be careful with their health and take a COVID-19 test if they have symptoms than younger people as reflected in the study findings. Younger people who are male, less educated, have lower income, who pay less attention or knew very little about COVID-19 were more likely to take proactive measures against COVID-19 infection [45]. Young people are also more likely to have asymptomatic or develop mild, transient illness making it possible to ignore the symptoms as it may not disturb their routine lives [46]. This study finding therefore aligns with prior observations.

Prior studies had also indicated that men are more vulnerable to COVID-19 than women [47] and have an increased risk of dying from COVID-19 than women [48]. This sex difference in the risk to COVID-19 infection and related mortality had been explained biologically. Females have more immune related genes responsible for boosting the innate immunity due to the double concentration of X chromosome [49]. Females therefore produce twice the quantity of antibodies and immunoglobulin G antigen in response to infection or vaccination [50], and develop more type 1 interferon, an efficient antiviral cytokine against COVID-19 viral infection [51]. Also, testosterone induces suppressive effect on immune system unlike the positive effect of oestrogen [52]. In addition, the presence of higher amount of angiotensin-converting enzyme 2 protein receptors—SARS-COV-2 mainly attack cells via these receptors—in males [53], their lower CD4 + T cell counts, vulnerable CD8 + T cell cytotoxic activity, and a decrease in formation of immunoglobulins by B cells in comparison to females [54, 55] all increase the vulnerability of men to COVID-19.

The regional difference in COVID–19 status is also important. This study provides evidence to suggest that the surveillance system instituted in Southern African may be more effective in promptly identifying and ensuring access of people with COVID-19 symptoms to testing when compared with other regions in sub-Saharan Africa. The surveillance system may however, have not been sufficient enough to mitigate and prevent COVID-19 transmission [56] as indicated by the high number of cases reported in the region [57]. Lessons can be learnt from the Southern Africa’s COVID-19 surveillance system to improve the surveillance system in other regions of sub-Saharan Africa taking cognisance of the context specific implementation differences [58].

Our study provides some insight into behavioural reasons that may explain these gender differences in the COVID-19 epidemiological profile: men were less likely to take a COVID-19 test when they have symptoms thereby delaying diagnosis and increasing the risk for severe diseases. This is consistent across various health conditions and comparable to previous findings in the region that have indicated that men are less likely to take a HIV test than women even when they have symptoms [59]. It is important to explore how gender norms related to masculinity may play a critical role in low COVID-19 testing in the region; and how perceptions and enactment of masculinity may contribute to men’s COVID-19 testing status in sub-Saharan Africa like it did with HIV testing in the region [59].

The study findings also have implications for COVID-19 public health response in the sub-Saharan Africa. The region has the highest burden of those living with HIV, the highest rate of mortality associated with HIV infection and high donor investment in HIV treatment programs for people living with HIV. It is therefore pertinent that programmes addressing the needs of people living with HIV should address barriers and challenges with uptake of COVID-19 tests when symptomatic to reduce the risk of mortality. The surveillance system also need to be strengthened to be cost effective by reaching out to all persons who tested positive to or died of COVID-19 infection. It is likely that the positivity yield will be much higher through this targeted testing. A surveillance system using this targeted testing may be more effective in timely containment of the pandemic in future. Strategic actions also need to be taken to identify and support people who are depressed and who have respiratory conditions to get tested. Future studies are needed to understand the challenges associated with and the barriers to accessing COVID-19 tests by those people who are depressed and who have respiratory conditions so as to design effective mitigations strategies.

## Conclusion

The findings suggest that residents of sub-Saharan Africa who had non-communicable diseases did not have a higher risk of testing positive for COVID-19. However, people at risk of COVID-19 infection and COVID-19 related deaths, including individuals with respiratory conditions and depression, were more likely to have symptoms of COVID-19 infection without getting tested. There are also indications that the COVID-19 surveillance system in the region is poor though the system in the Southern Africa sub region may be stronger than that in other sub regions. Finally, compared with those who live alone, people who cohabitate appear to take more COVID-19 precautionary measures to reduce their risk of contracting COVID-19 infection. Therefore, future research should further explore the motivations behind health-related decision-making practices among various at-risk populations to inform new regional-specific and population-targeted public health campaigns.

## Supplementary Information

**Additional file 1.** List of countries and number of respondents from the country. This file contains the list of the 31 countries in sub-Saharan Africa from where study participants were recruited. It also contains details on the number and percentage of participants recruited from these countries.

**Additional file 2.** Study Questionnaire. This is the complete questionnaire used to collect data for the study.

**Additional file 3.** Binary logistic regression separating Eastern and Southern Africa. This file contains details of the outcome of a Binary logistic regression to determine factors the association between HIV testing status, COVID-19 status non-communicable disease and HIV status for residents of sub-Saharan Africa. It is a supplemental analysis that included Eastern and Southern Africa as independent variables.

## Data Availability

The datasets used and/or analysed during the current study are available from the corresponding author on reasonable request.

## Abbreviations

AOR: Adjusted odds ratio
COVID-19: Corona Virus Disease 2019
HIV: Human immunodeficiency virus
