# Supplementary material for: Associations between COVID-19 testing status, non-communicable diseases and HIV status among residents of sub-Saharan Africa during the first wave of the pandemic
Source: BMC Infect Dis. 2022 Jun 13;22:535. doi: 10.1186/s12879-022-07498-w (PMC9188915; doi:10.1186/s12879-022-07498-w)
Supplement: Supplementary file 1 — Additional file 1. List of countries and number of respondents from the country. This file contains the list of the 31 countries in sub-Saharan Africa from where study participants were recruited. It also contains details on the number and percentage of participants recruited from these countries. [file 12879_2022_7498_MOESM1_ESM.docx]

Supplemental file 1: List of countries and number of respondents from the country

| **Serial no** | **Country** | **Frequency** | **Percentage** |  |
| --- | --- | --- | --- | --- |
| 1 | Cameroon | 12 | 0.2 | Central Africa |
| 2 | Congo, Democratic Republic of the | 5 | 0.1 | Central Africa |
| 3 | Congo, Republic of the | 19 | 0.3 | Central Africa |
| 4 | Ethiopia | 7 | 0.1 | Eastern Africa |
| 5 | Mauritius | 7 | 0.1 | Eastern Africa |
| 6 | Rwanda | 15 | 0.3 | Eastern Africa |
| 7 | South Sudan | 4 | 0.1 | Eastern Africa |
| 8 | Kenya | 38 | 0.6 | Eastern Africa |
| 9 | Tanzania | 21 | 0.4 | Eastern Africa |
| 10 | Uganda | 65 | 1.1 | Eastern Africa |
| 11 | Malawi | 20 | 0.3 | Southern Africa |
| 12 | Namibia | 22 | 0.4 | Southern Africa |
| 13 | Angola | 3 | 0.1 | Southern Africa |
| 14 | Botswana | 28 | 0.5 | Southern Africa |
| 15 | Eswatini (formerly Swaziland) | 5 | 0.1 | Southern Africa |
| 16 | Lesotho | 2 | 0.0 | Southern Africa |
| 17 | South Africa | 572 | 9.6 | Southern Africa |
| 18 | Zambia | 4 | 0.1 | Southern Africa |
| 19 | Zimbabwe | 46 | 0.8 | Southern Africa |
| 20 | Mauritania | 1 | 0.0 | Western Africa |
| 21 | Benin | 12 | 0.2 | Western Africa |
| 22 | Burkina Faso | 3 | 0.1 | Western Africa |
| 23 | Cote d'Ivoire | 32 | 0.5 | Western Africa |
| 24 | Gambia | 9 | 0.2 | Western Africa |
| 25 | Ghana | 363 | 6.1 | Western Africa |
| 26 | Liberia | 18 | 0.3 | Western Africa |
| 27 | Mali | 31 | 0.5 | Western Africa |
| 28 | Nigeria | 4563 | 76.8 | Western Africa |
| 29 | Senegal | 10 | 0.2 | Western Africa |
| 30 | Sierra Leone | 7 | 0.1 | Western Africa |
| 31 | Togo | 1 | 0.0 | Western Africa |
|  | **Total** | **5945** | **100.0** |  |
