# Supplementary material for: Associations between COVID-19 testing status, non-communicable diseases and HIV status among residents of sub-Saharan Africa during the first wave of the pandemic
Source: BMC Infect Dis. 2022 Jun 13;22:535. doi: 10.1186/s12879-022-07498-w (PMC9188915; doi:10.1186/s12879-022-07498-w)
Supplement: Supplementary file 2 — Additional file 2. Study Questionnaire. This is the complete questionnaire used to collect data for the study. [file 12879_2022_7498_MOESM2_ESM.pdf]

You are invited to complete an online survey which aims to explore the impact that the COVID-19 outbreak has on the wellness and wellbeing of people. The research is based at the Obafemi Awolowo University Ile-Ife and Lead by Morenike Oluwatoyin Folayan. Ethical approval was obtained from the Human Research Ethics Committee at Institute of Public Health of the Obafemi Awolowo University Ile-Ife, Nigeria (HREC No: IPHOAU/12/1557). The list of co-investigators are found at the end of this consent form.

You have been invited to take part in this project because you are fluent in speaking and understanding the English language, aged 18 years or older and have access to the online survey. Learning from you about how the COVID-19 pandemic has affected the people's wellness and well-being of people of different diversities will help us make recommendations that may improve considerations for people's wellness and well-being in this and future pandemics.

You are under no obligation to take part in this survey. If you would like to participate in the survey, then you are required to answer the questions in the Consent section. This will allow you to access and answer the survey questions. You can end your participation at any time by exiting the survey or closing the web-browser. The withdrawal of any data that you provide is not possible once you have commenced the online survey.

The survey will take about 8-10 minutes to complete the survey. The data collected from the online survey will be kept anonymously and confidentially in a secure setting. It will only be accessed by the research team.

If you are unhappy with any aspects of the research, or if there is a problem, please let us know by contacting

Prof Morenike Oluwatoyin Folayan, Study lead, Obafemi Awolowo University, Ile-Ife, **toyinukpong@yahoo.co.uk**

Dr Brandon Brown, University of California, Riverside School of Medicine, Riverside, California, USA

Dr Annie Nguyen, University of Southern California, Los Angeles, California.

Prof Oliver Ezechi, Nigeria Institute of Medical Research, Nigeria

Prof Benjamin Uzochukwu, University of Nigeria, Enugu Campus, Nigeria

Prof Maha El Tantawi, Alexandria University, Alexandria, Egypt

Dr Nourhan M Aly, Alexandria University, Alexandria, Egypt

Consent Page

* 1. I confirm that I am aged 18 years or older

- Yes
- No

* 2. I confirm that I have read and have understood the Participant Information Sheet for the current study. I have had the opportunity to consider the information, ask questions and have had these answered satisfactorily

- Yes
- No

* 3. I understand that my participation is voluntary and that I am free to end my participation at any time by closing the survey or web-browser without giving any reason.

- Yes
- No

* 4. I understand that the withdrawal of any data that I provide is not possible once I have commenced the online survey

- Yes
- No

* 5. I agree to take part in this survey

- Yes
- No

COVID-19 Information

* 6. I have tested positive for COVID-19

◯ Yes

◯ No

* 7. I have experienced the symptoms of COVID-19 (persistent cough or high body temperature or loss of smell) since the outbreak began but I was not tested

◯ Yes

◯ No

* 8. I have a close friend who tested positive for COVID-19

◯ Yes

◯ No

* 9. I know someone who died from COVID-19

◯ Yes

◯ No

* 10. I have had to self-isolate because I have symptoms of COVID-19 before or after the ‘lockdown’

◯ Yes

◯ No

* 11. Have you taken at least one dose of a COVID-19 vaccine?

◯ Yes

◯ No

If your answer to the previous question was **No**, please answer the following two questions

12. Are you willing to receive a COVID-19 vaccine?

◯ Yes

◯ No

◯ Unsure

13. What is the reason you have not taken a COVID-19 vaccine? (select all that apply)

- [ ] I do not have access because it is not available in my country
- [ ] I do not have access because vaccination facilities are very crowded
- [ ] I do not have access because I have to wait for my turn for quite some time
- [ ] I cannot afford it
- [ ] I do not have enough information about the vaccine
- [ ] I am concerned the vaccine is not safe
- [ ] I am concerned about taking more than one dose
- [ ] I am unsure that it will work
- [ ] I do not trust the companies making the vaccine
- [ ] My religion or beliefs prevent me from taking one
- [ ] I have already been infected with COVID-19
- [ ] I am at low-risk for COVID-19
- [ ] The vaccine side effects match the disease effects
- [ ] I do not want to be forced to take the vaccine
- [ ] The vaccine is part of governments' plots to control people
- [ ] I am taking all the needed precautions so I do not need it
- [ ] None of the vaccines are adequately studied; still too early

Section 1: Socio-demographic Profile

* 14. In what year were you born?

* 15. What is your country of residence?

* 16. What is the highest level of education you completed?

- No formal education
- Primary
- Secondary
- University
- Post-graduate

* 17. What is your current work status?

- Employed full time
- Employed part time
- Self-employed
- Unemployed looking for work
- Unemployed not looking for work
- Unemployed but volunteering
- Retired
- Student
- Family/Home carer

* 18. Are you a healthcare worker?

- Yes
- No

* 19. Do you have medical insurance?

- Yes, public insurance
- Yes, private insurance
- No

* 20. Who do you currently live with?

- By myself
- With a spouse or partner
- With related family members
- With people who are not related to me

* 21. What is your current relationship status?

- ◯ Single
- ◯ Legally married
- ◯ Co-habiting
- ◯ Divorced/separated
- ◯ Widowed

* 22. What sex were you assigned at birth? (For example listed on your birth certificate)

- ◯ Male
- ◯ Female
- ◯ Intersex
- ◯ Decline to answer

* 23. What is your current gender?

- ◯ Man
- ◯ Transgender woman/male-to-female
- ◯ Woman
- ◯ Gender nonconforming
- ◯ Transgender man/female-to-male
- ◯ Decline to answer
- ◯ Other (Please specify)

____

* 24. What is your sexual orientation?

- ◯ Straight or heterosexual
- ◯ Lesbian or gay
- ◯ Bisexual
- ◯ Decline to answer
- ◯ Other (please specify)

____

* 25. Do you engage in any of the following behaviors? (choose all that applies)

- ☐ Engage in transactional sex
- ☐ Use illegal drugs
- ☐ Use prescription drugs without a prescription
- ☐ Inject drugs using a needle
- ☐ Not applicable

Section 2: Medical Health Status

* 26. Please indicate if you have any of the following conditions (check all that apply)

- [ ] None
- [ ] Anxiety
- [ ] Arthritis
- [ ] Asthma
- [ ] Broken bones or fractures
- [ ] Cancer
- [ ] Depression
- [ ] Dermatological problems
- [ ] Diabetes
- [ ] Difficulty eating, bathing, dressing, toileting, or moving around by yourself
- [ ] Hearing loss
- [ ] Heart condition
- [ ] Hepatitis
- [ ] Herpes - Herpes simplex
- [ ] High blood pressure
- [ ] HIV
- [ ] Kidney conditions
- [ ] Malaria
- [ ] Migraines
- [ ] Neurological problems
- [ ] Pneumonia
- [ ] Respiratory problems
- [ ] Severe allergy
- [ ] Shingles - Herpes Zoster
- [ ] STI (sexually transmitted infections such as gonorrhea, syphilis, clamydia)
- [ ] Stroke
- [ ] Urinary tract infections
- [ ] Vision loss
- [ ] (Others) Please specify

* 27. As compared to 5 years ago, how would you describe your ability to perform the following tasks involving your memory?

	Much better now	Somewhat better now	About the same	Somewhat poorer now	Much poorer now
Remembering the name of the person just introduced to you	○	○	○	○	○
Recalling a radio/television station or zip code number that you use on a daily or weekly basis	○	○	○	○	○
Recalling where you have put objects (such as keys) in your home	○	○	○	○	○
Remembering specific facts from an internet article, newspaper or magazine article you have just finished reading	○	○	○	○	○
Remembering the items you intended to buy when you arrive at a shop or pharmacy	○	○	○	○	○
In general, how would you describe your memory as compared to 5 years ago?	○	○	○	○	○

Section 3: Pandemic Stress Index

Many countries issued a complete or partial restricted movement order in response to the COVID-19 (coronavirus) pandemic. The following questions are about the time period that these orders were in place.

* 28. Which of the following are you doing during COVID-19 ? (check all that apply)

- [ ] I made no changes to my life or behavior
- [ ] Practicing physical distancing (i.e., reducing physical contact with other people in social, work, or school settings by avoiding large groups and staying 6 feet away from other people)
- [ ] Isolating or quarantining myself (i.e., because you were sick, or you were exposed to coronavirus and separated yourself from other people to prevent others from getting it)
- [ ] Wearing masks or face coverings
- [ ] Washing my hands or sanitizing my hands more often
- [ ] Caring for someone at home
- [ ] Working from home
- [ ] Volunteering my time, skills, resources, or donating money to help fight COVID-19
- [ ] Following media coverage related to COVID-19 (e.g., watching or reading the news, following social media coverage, etc.)
- [ ] Change in work status
- [ ] Change in use of healthcare services
- [ ] Change in travel plans

* 29. How much did COVID-19 impact your day-to-day life?

- ○ Extremely
- ○ Very much
- ○ Much
- ○ A little
- ○ Not at all

* 30. Which of the following are you experiencing during this COVID-19 period? (check all that apply)

- [ ] I have been diagnosed with COVID-19
- [ ] Fear of getting COVID-19
- [ ] Fear of giving COVID-19 to someone else
- [ ] Worrying about people other than me (friends, family, partners, etc.)
- [ ] Stigma or discrimination from other people (e.g., people treating you differently because of your identity, having symptoms, or other factors related to COVID-19)
- [ ] Frustration or boredom
- [ ] Anxiety
- [ ] Depression
- [ ] Loneliness
- [ ] Anger
- [ ] Grief or feelings of loss
- [ ] Change in sleep patterns (sleeping more, sleeping less, or other changes from usual)
- [ ] Confusion about what COVID-19 is, how to prevent it, or why social distancing/isolation/quarantines are needed
- [ ] Feeling that I was contributing to the greater good by preventing myself or others from getting COVID-19
- [ ] Not getting enough emotional or social support from family, friends, partners, a counselor, or someone else
- [ ] Not getting enough financial support from family, friends, partners, an organization, or someone else
- [ ] Not getting enough exercise
- [ ] Confusion about where to get true and accurate information about COVID-19
- [ ] Difficulty obtaining a mask or face covering
- [ ] Difficulty washing my hands as often as recommended
- [ ] None of the above

* 31. Over the last 7 days, how often have you been bothered by the following problems:

	Not at all	Several days	More than half the days	Nearly every day
Having little interest or pleasure in doing things?	○	○	○	○
Feeling down, depressed, or hopeless?	○	○	○	○
Feeling nervous, anxious, or on edge?	○	○	○	○
Not being able to stop or control worrying?	○	○	○	○

* 32. In the past 6 months, how often have you thought about taking your own life

- ○ Never
- ○ Seldom
- ○ Very often
- ○ All the time

If you are thinking about suicide, be sure to talk to other people about it. They can be friends or family members but they do not have to be. It can be difficult to talk about this topic with people close to you. What is important is that you talk to someone. You can do this by phone, chat, email or in person.

Section 3: Pandemic Stress Index

* 33. What kind of "Change in work status"did you face?

- ◯ Work was laid off
- ◯ Reduced working hours
- ◯ Increased working hours
- ◯ Other (Please specify)

* 34. In case of "Change in use of healthcare services". Was this an increase or decrease in use of services?

- ◯ Increase
- ◯ Decrease

* 35. What kind of "Change in travel plans"did you face?

- ◯ Cancelled travel plans
- ◯ Scheduling more travel plans
- ◯ Others (Please specify)

Section 4: Finance and Lifestyle

* 36. Have you experienced a change in...

	Increase	Decrease	No change	Not applicable
Sexual activity	◯	◯	◯	◯
Tobacco use	◯	◯	◯	◯
Alcohol use	◯	◯	◯	◯
Marijuana use	◯	◯	◯	◯
Other substance use	◯	◯	◯	◯
Food intake	◯	◯	◯	◯
Use of screens [computers, smart phones: Internet addiction and gaming mostly]	◯	◯	◯	◯

* 37. Have you experienced a financial loss because of the COVID-19 pandemic?

◯ Yes

◯ No

◯ Not sure

Section 4: Finance and Lifestyle

* 38. What financial losses have you experienced because of the COVID-19 pandemic? (check all that apply)

- [ ] Job loss or laid off
- [ ] Lost or reduced wages
- [ ] Investment/retirement loss
- [ ] Travel-related cancellations that were not refunded
- [ ] Other (Please specify)

* 39. Has the COVID-19 pandemic led to any of the following?

	Yes	No
Having to spend more time taking care of partners or other family members	◯	◯
Loss of other sources of financial support by you or a member of your household	◯	◯
Loss of your housing, or becoming homeless	◯	◯
Difficulty paying for basic needs, including food, clothing, shelter, electricity, utilities, etc.	◯	◯
Did you worry about whether food would run out before getting money to buy more?	◯	◯
Did you ever cut the size of your meals or skip meals because there wasn't enough money for food?	◯	◯
Were you ever hungry but didn't eat because there wasn't enough money for food?	◯	◯
Unable to attend a healthcare providers appointment	◯	◯
Unable to obtain medications that you take	◯	◯
Unable to afford medical care	◯	◯

Section 4: Finance and Lifestyle

* 40. Did your toothbrushing frequency change?

- Yes, increased
- Yes, decreased
- No, did not change

* 41. Did you have mouth ulcers during the lockdown?

- Yes
- No

* 42. Did you have critical medical need during the COVID-19 Pandemic?

- Yes
- No
- No response

* 43. Did you have challenges accessing usual medical health care services?

- Yes
- No
- No response
- Not applicable

* 44. Did you have to resort to alternative medical care services to address your health needs?

- Yes
- No
- No response
- Not applicable

* 45. Did you have a healthcare provider appointment at any time during the COVID-19 pandemic that you were not able to attend?

- Yes
- No
- I did not have a healthcare provider appointment during COVID-19

Section 4: Finance and Lifestyle

* 46. What were the reasons that made you unable to attend a healthcare provider's appointment? (check all that apply)

- [ ] My clinic cancelled my appointment because of COVID-19
- [ ] Hours/location was inconvenient
- [ ] I had symptoms of coronavirus so did not go
- [ ] I forgot to go/missed my appointment
- [ ] I felt good so did not need to go
- [ ] I felt disrespected by the office or medical staff
- [ ] Didn't have the money or insurance
- [ ] I had difficulty getting transportation to the clinic
- [ ] Other (Please specify)

Section 4: Finance and Lifestyle

* 47. How much has the COVID-19 pandemic interrupted the care you receive for your mental health? (e.g., counselor, therapist, support groups?)

- ◯ I don’t receive mental health care
- ◯ Not at all
- ◯ Somewhat
- ◯ A lot

* 48. How much has the COVID-19 pandemic interrupted the care you receive for substance abuse addiction?

- ◯ I don’t receive substance abuse addiction care
- ◯ Not at all
- ◯ Somewhat
- ◯ A lot

* 49. If you used any healthcare service at all during the COVID-19 pandemic, how did you meet your provider? (Check all that apply)

- ☐ In-person visit
- ☐ Telehealth with video
- ☐ Over the phone (no video)
- ☐ Email or written communication (e.g., through a patient portal)
- ☐ Not applicable

Section 5: Psychosocial Support

* 50. On a scale from 1 (lowest) to 10 (highest), how socially isolated do you feel right now?

0 — 10 [ ]

* 51. Compared to your life before COVID-19, do you feel...

- More socially isolated
- Less socially isolated
- About the same/ no change

* 52. How much difficulty have you had following the recommendations for keeping away from close contact with people during COVID-19?

- None
- A little
- Moderate
- A lot

* 53. How has the quality of the relationships between you and members of your family changed?

- A lot worse
- A little worse
- About the same
- A little better
- A lot better
- Not applicable

* 54. How has the quality of the relationships between you and your significant other changed?

- A lot worse
- A little worse
- About the same
- A little better
- A lot better
- Not applicable

* 55. How has the quality of the relationships with your friends changed?

- A lot worse
- A little worse
- About the same
- A little better
- A lot better
- Not applicable

Section 6: Post-Traumatic Stress Disorder

* 56. Below is a list of problems and complaints that people sometimes have in response to stressful life experiences. Please read each one carefully, then check off to the right to indicate how much you have been bothered by that problem in the past month.

	Not at all	A little bit	Moderately	Quite a bit	Extremely
Repeated, disturbing memories, thoughts, or images of a stressful experience from the past?	○	○	○	○	○
Repeated, disturbing dreams of a stressful experience from the past?	○	○	○	○	○
Suddenly acting or feeling as if a stressful experience were happening again (as if you were reliving it)?	○	○	○	○	○
Feeling very upset when something reminded you of a stressful experience from the past?	○	○	○	○	○
Having physical reactions (e.g., heart pounding, trouble breathing, sweating) when something reminded you of a stressful experience from the past?	○	○	○	○	○
Avoiding thinking about or talking about a stressful experience from the past or avoiding having feelings related to it?	○	○	○	○	○
Avoiding activities or situations because they reminded you of a stressful experience from the past?	○	○	○	○	○
Trouble remembering important parts of a stressful experience from the past?	○	○	○	○	○

	Not at all	A little bit	Moderately	Quite a bit	Extremely
Loss of interest in activities that you used to enjoy?	○	○	○	○	○
Feeling distant or cut off from other people?	○	○	○	○	○
Feeling emotional, numb, or being unable to have loving feelings for those close to you?	○	○	○	○	○
Feeling as if your future will somehow be cut short?	○	○	○	○	○
Trouble falling or staying asleep?	○	○	○	○	○
Feeling irritable or having angry outbursts?	○	○	○	○	○
Having difficulty concentrating?	○	○	○	○	○
Being “super-alert” or watchful or on guard?	○	○	○	○	○
Feeling jumpy or easily startled?	○	○	○	○	○

Section 7: Coping

* 57. These items deal with ways you have been coping with the stress in your life since the onset of the COVID-19 pandemic. Consider how well the following statements describe your behavior and actions.

	Does not describe me at all	Does not describe me	Neutral	Describes me	Describes me very well
I look for creative ways to alter difficult situations.	○	○	○	○	○
Regardless of what happens to me, I believe I can control my reaction to it.	○	○	○	○	○
I believe I can grow in positive ways by dealing with difficult situations.	○	○	○	○	○

Section 8: Self Care

* 58. What are the things you have done to take care of your mental health during the COVID-19 pandemic? (select all that apply)

- [ ] Talk to friends or family on the phone
- [ ] Talk to friends or family through videochat (Facetime, Skype, Zoom, Line, WhatsApp, Viber etc.)
- [ ] Talk to friends or family face to face, in person
- [ ] Spend time with pets
- [ ] Meditate or other mindfulness practices
- [ ] Exercise in or around your home
- [ ] Other (Please specify)
- [ ] Exercise or spend leisure time outdoors like at a park or walking trail
- [ ] Do yardwork or gardening
- [ ] Participate in creative activities or hobbies (writing, reading, art, crafts)
- [ ] Learn a new skill or engage in distant learning
- [ ] Taking breaks from the news or social media

* 59. How did you learn about this study?

- ○ Facebook
- ○ WhatsApp
- ○ Twitter
- ○ LinkedIn
- ○ Website
- ○ Personal invitation

Other (please specify)

60. Are there other challenges you have faced during the COVID-19 pandemic that we did not ask about?

61. Are there other strengths or resiliencies that you have tapped into that we did not ask about? Please describe here:

Section 9: For People Living with HIV

* 62. What was the year of your first HIV positive test?

* 63. What is your viral load?
   - Detectable
   - Undetectable
   - Do not know

64. If you know your viral load, how many copies per ml? (e.g., 20 copies/mL)

* 65. What was your most recent CD4 count?
   - Less than 200
   - Between 200 and 500
   - More than 500
   - Do not know

* 66. What was the lowest CD4 count you’ve ever had (nadir CD4)?
   - Less than 200
   - Between 200 and 500
   - More than 500
   - Do not know

* 67. At this moment, do you have a 90-day supply of your HIV medications?
   - Yes
   - No

* 68. Did you have difficulty obtaining a 90-day supply of HIV medications during COVID-19?
   - Yes
   - No

* 69. Some people find that they sometimes forget to take their medications to manage their HIV. Did you miss any of your HIV medications during COVID-19?

- Yes
- No

Section 9: For People Living with HIV

* 70. What are the reasons you did not take your HIV medications? (select all that apply)

- ☐ Could not get my HIV medications because pharmacy was closed or because of the shutdowns
- ☐ Wanted to ration my medication to make it last during the shutdowns
- ☐ Felt good, did not need them
- ☐ CD4 count and viral load are good
- ☐ Doctor advised me to delay treatment
- ☐ Don't want to think about being HIV positive
- ☐ Other (Please specify)
- ☐ Worried about side effects
- ☐ Don't have money or insurance to get medicine
- ☐ Drinking or using drugs and did not want interactions
- ☐ I just recently found out I was HIV positive
- ☐ I forgot to take them

71. Thank you for taking this survey.

If you will like us to contact you with the publications associated with this study, please provide me your email address

The Survey Code is: KXJK-87XB-UZKU
