# Supplementary material for: Associations between COVID-19 testing status, non-communicable diseases and HIV status among residents of sub-Saharan Africa during the first wave of the pandemic
Source: BMC Infect Dis. 2022 Jun 13;22:535. doi: 10.1186/s12879-022-07498-w (PMC9188915; doi:10.1186/s12879-022-07498-w)
Supplement: Supplementary file 3 — Additional file 3. Binary logistic regression separating Eastern and Southern Africa. This file contains details of the outcome of a Binary logistic regression to determine factors the association between HIV testing status, COVID-19 status non-communicable disease and HIV status for residents of sub-Saharan Africa. It is a supplemental analysis that included Eastern and Southern Africa as independent variables. [file 12879_2022_7498_MOESM3_ESM.docx]

Supplemental File 1: Binary logistic regression to determine factors the association between HIV testing status, COVID-19 status non-communicable disease and HIV status for residents of sub-Saharan Africa (N=5945)

|  |  |  |  |  |  |  |  |  |
| --- | --- | --- | --- | --- | --- | --- | --- | --- |
| **Variables** | **Tested positive for COVID-19 infection** | | | | **Had symptoms of COVID-19 but did not get tested** | | | |
|  | AOR | 95% C.I. for AOR | | P value | AOR | 95% C.I. for AOR | | P value |
|  |  | Lower | Upper |  |  | Lower | Upper |  |
| **Age** | 0.992 | 0.974 | 1.010 | 0.364 | 0.955 | 0.945 | 0.966 | <0.001 |
| **Males (Ref: females)** | 0.993 | 0.718 | 1.373 | 0.967 | 1.374 | 1.157 | 1.632 | <0.001 |
| **Education (Ref: No formal education)** | 1. 000 | **-** | **-** | **-** | 1. 000 | **-** | **-** | **-** |
| Primary | 0.884 | 0.107 | 7.327 | 0.909 | 0.688 | 0.212 | 2.238 | 0.535 |
| Secondary | 0.458 | 0.086 | 2.433 | 0.359 | 0.936 | 0.367 | 2.385 | 0.889 |
| Tertiary (University) | 0.291 | 0.057 | 1.480 | 0.137 | 0.921 | 0.367 | 2.314 | 0.861 |
| **Employment status (Ref: unemployed)** | 1. 000 | **-** | **-** | **-** | 1. 000 | **-** | **-** | **-** |
| Retired | 2.295 | 0.697 | 7.554 | 0.172 | 1.345 | 0.564 | 3.211 | 0.504 |
| Students | 0.557 | 0.259 | 1.196 | 0.133 | 0.892 | 0.646 | 1.233 | 0.489 |
| Employed | 0.998 | 0.589 | 1.691 | 0.995 | 0.990 | 0.772 | 1.270 | 0.938 |
| **Sub region** |  |  |  |  |  |  |  |  |
| Western and Central Africa | 1. 000 | **-** | **-** | **-** | 1. 000 | **-** | **-** | **-** |
| Eastern Africa | 0.486 | 0.150 | 1.579 | 0.230 | 0.885 | 0.514 | 1.522 | 0.658 |
| Southern Africa | 0.885 | 0.569 | 1.375 | 0.587 | 0.738 | 0.567 | 0.960 | 0.023 |
| **Medical health profile** |  |  |  |  |  |  |  |  |
| Diabetes (ref: no) | 0.660 | 0.223 | 1.958 | 0.454 | 1.124 | 0.616 | 2.051 | 0.703 |
| Hypertension (ref: no) | 1.566 | 0.926 | 2.650 | 0.095 | 1.203 | 0.850 | 1.702 | 0.297 |
| Cancer (ref: no) | 0.687 | 0.072 | 6.570 | 0.745 | 1.329 | 0.322 | 5.485 | 0.694 |
| Heart condition (ref: no) | 1.632 | 0.515 | 5.166 | 0.405 | 1.942 | 0.881 | 4.277 | 0.100 |
| Respiratory condition (ref: no) | 0.824 | 0.192 | 3.549 | 0.796 | 2.485 | 1.347 | 4.587 | 0.004 |
| Depression (ref: no) | 1.095 | 0.606 | 1.978 | 0.764 | 1.909 | 1.447 | 2.519 | <0.001 |
| **Living with HIV (ref: no)** | 0.287 | 0.131 | 0.630 | 0.002 | 1.278 | 0.979 | 1.666 | 0.071 |
| **COVID-19 status** |  |  |  |  |  |  |  |  |
| I have a close friend who tested positive for COVID-19 (ref: no) | 6.710 | 4.704 | 9.573 | <0.001 | 2.568 | 2.117 | 3.114 | <0.001 |
| I know someone who died from COVID-19  (ref: no) | 1.733 | 1.232 | 2.438 | 0.002 | 1.813 | 1.511 | 2.175 | <0.001 |
| **Cohabiting (Ref: Yes)** | 1.513 | 1.058 | 2.163 | 0.025 | 1.020 | 0.833 | 1.249 | 0.849 |
|  |  |  |  |  |  |  |  |  |
|  |  |  |  |  |  |  |  |  |
